# Web-Based Tool for Australian Family Day Care to Promote Healthy Lifestyles: Randomized Controlled Trial

**DOI:** 10.1177/15248399251328360

**Published:** 2025-03-30

**Authors:** Georgie Tran, Bridget Kelly, Sarah T. Ryan, Megan Hammersley, Erin Kerr, Jennifer Norman, Mel Leedham, Cecilia Vuong, Karen Wardle, Anthony Okely

**Affiliations:** 1 University of Wollongong, Wollongong, New South Wales, Australia; 2 Sydney Local Health District, Camperdown, New South Wales, Australia; 3 Illawarra Shoalhaven Local Health District, Warrawong, New South Wales, Australia; 4 South Western Sydney Local Health District, Liverpool, New South Wales, Australia

**Keywords:** family day care, quality improvement, health education, professional development, nutrition, physical activity

## Abstract

Food and physical activity environments in family day care can be improved to better support healthy behaviors. A 6-month two-arm parallel randomized controlled trial evaluated the effectiveness of a web-based tool to promote healthier practices through quality improvement planning among Australian service providers and their educators. Service providers were randomized 1:1 into the intervention group (using the tool for a minimum of 1 month alongside regular quality improvement plan processes) and control group (regular quality improvement plan processes). The primary outcome of change in the quality of the improvement plan (in relation to the incorporation of healthy practices) was assessed using a checklist designed specifically for the study. Secondary outcomes were self-ratings of awareness and knowledge of various topics assessed using a 5-point Likert-type scale. Eight service providers and 22 educators participated (four service providers and 10 educators in the intervention group; four service providers and 12 educators in the control group). Intention-to-treat analyses found significant change in quality of the revised improvement plan for the intervention group. The intervention group showed an increase in self-rated awareness and knowledge on healthy practices and National Quality Standards, and confidence in identifying priority areas. This is the first-known web-based tool designed specifically for family day care to promote healthier practices. There are several opportunities for the tool to be embedded in practice, including delivery of the tool as part of support programs or training. This trial is registered with Australian New Zealand Clinical Trials Registry (ANZCTR) ACTRN12623000369628.

F

amily day care is an important setting to promote children’s healthy behaviors (Wallace & Mills, 2019). In Australia, family day care is part of early childhood education and care, serving 5.1% of the 1,419,380 children who regularly attend child care (Department of Education Australian Government, 2024). Research suggests that food and physical activity environments in family day care can be improved (Daniels et al., 2003; Kerr et al., 2021; Wallace & Mills, 2019).

In New South Wales Australia, family day care service providers oversee operations of care in educators’ homes (Ishimine & Tayler, 2012). Service providers must have a quality improvement plan (QIP) to assess performance, plan future actions, and demonstrate that they have met National Quality Standards (NQS) (Australian Children’s Education & Care Quality Authority, 2018). QIPs must be reviewed annually (Australian Children’s Education & Care Quality Authority, 2018).

Nutrition and physical activity practices are referenced in Quality Area 2 of the NQS, which specifies that healthy eating and physical activity be promoted and tailored to each child (Australian Children’s Education & Care Quality Authority, 2018). There is guidance from the Australian Government’s *Get Up & Grow* guidelines (Australian Government Department of Health and Aged Care, n.d.) and state initiatives like New South Wales “*Munch & Move*” program, which encourages and supports child care services to implement healthy eating and physical activity strategies (New South Wales Government, 2020).

A web-based tool, the *Family Day Care Quality Improvement Toolkit* (the Toolkit), was co-developed in 2022 with family day care stakeholders to assist service providers in reviewing QIPs while incorporating nutrition and physical activity practice improvements. Formative evaluation showed that the Toolkit was feasible, easy to use, and relevant to practice (Tran et al., 2025).

This study aimed to evaluate the Toolkit’s acceptability and assess the extent to which nutrition and physical activity practices are incorporated into quality improvement planning.

## Methods

### Study Design

This study was registered with Australian New Zealand Clinical Trials Registry ACTRN12623000369628 (Australian New Zealand Clinical Trials Registry, 2023). It was a two-arm parallel randomized controlled trial conducted in June to December 2023 in New South Wales, Australia. This study followed the CONSORT-EHEALTH statement (Eysenbach & Group, 2011) and received approval from the University of Wollongong Human Research Ethics Committee (2023/PID00230). Online informed consent was obtained from all participants.

### Participants

Recruitment ran for 6 months to align with various QIP review timeframes. New South Wales Local Health District health promotion staff, funded to work with service providers, assisted by forwarding email invitations and including them in newsletters. Direct online recruitment (emails and calls) was conducted using a publicly available list from the Australian Children’s Education and Care Quality Authority (ACECQA). The New South Wales Family Day Care Association also assisted in forwarding email invitations.

Interested service providers were asked to involve their educators, as per usual QIP review. As the number of educators registered with a service provider varied, so too did the number of educators who participated. At least one educator was required per service provider. Inclusion criteria included being 18 years or older, undergoing a QIP review (for service providers), and having English proficiency and internet/computer literacy. Participants provided online consent via REDCap version 14.0.15 (Harris et al., 2009), where baseline measures were also collected through an online survey. Measures included demographic details, self-ratings of awareness and knowledge of *Munch & Move* practices, the NQS, and perceived importance and confidence in promoting nutrition and physical activity. Service providers uploaded a digital copy of their previous QIP to the survey.

Service providers in the control group reviewed their QIP using their regular processes, without the Toolkit. After completing their review, they provided a digital copy of the revised QIP.

Participants in the intervention group used the Toolkit alongside their regular QIP review process, with access provided for a minimum of 1 month depending on how long they required access.

The Toolkit was designed by the University of Wollongong research team with input from an advisory group, including representatives from New South Wales Ministry of Health, Local Health Districts and family day care service providers and educators. Formative evaluation occurred in January to February 2023. The tool was finalized by April 2023 and hosted on a website with user login functionality. Service providers and educators accessed the same Toolkit; however, accessible features differed. The Toolkit’s content and design were informed by ACECQA QIP resources (Australian Children’s Education & Care Quality Authority, 2018).

No changes were made during the intervention. No formal training was required, as the tool guided the users through step-by-step instructions. It was not possible to specify the timing and frequency of usage as QIP review processes varied depending on internal processes. Email prompts were sent at the start and 1 week before the end of the intervention as reminders.

For educators, the Toolkit contained a self-assessment questionnaire, which was developed based on *Munch & Move* internal documents from New South Wales Ministry of Health. The questionnaire linked with the relevant NQS and *Munch & Move* key messages. If an educator did not receive the highest rating for a question, targeted multimedia *Munch & Move* resources were provided. There was also a self-assessment reflection component that aimed to guide educators on critical reflection of practices.

For service providers, there was a tailored dashboard with visual and written summaries of their educators’ performance, highlighting key priority areas related to *Munch & Move* practices. Service providers could then complete their required QIP review with the Toolkit’s “Action Plan” template, which was adapted from the Improvement Plan section of the ACECQA QIP template (Australian Children’s Education & Care Quality Authority, 2018). Supplementary file 1 provides screenshots of the login pages of the Toolkit.

### Measures

The primary outcome was change in the quality of the QIP (in relation to the incorporation of nutrition and physical activity practices) between baseline (the service’s previous QIP) and follow-up (revised QIP). The previous and revised QIPs were benchmarked against a checklist designed specifically for the study (Supplementary file 2). The checklist was composed of two sections: (1) QIP components assessed with binary outcomes (Yes—score = 1 / No—score = 0) and (2) alignment with *Munch & Move* practices assessed with a scoring system of 0 to 2.

The first checklist section was designed to align with the ACECQA QIP template to ensure accurate assessment of current QIP requirements. A total score out of 9 was calculated, with a higher score representing better alignment with NQS, development of actionable plans, identification of relevant priority areas, and incorporation of educators’ practices. The second checklist section was designed to align with *Munch & Move* practices to ensure nutrition and physical activity recommendations were addressed. A total score out of 8 was calculated, with a higher score representing that *Munch & Move* practice areas were mentioned in the QIP and aligned with good practice. For this section, a score of 0 indicated that the particular *Munch & Move* practice was not mentioned in the QIP nor aligned with good practice. A score of 1 indicated that the practice was mentioned but not fully aligned with good practice, and a score of 2 indicated that the practice was both mentioned and aligned with good practice. The highest score possible for the entire checklist was 17/17.

Secondary outcomes included qualitative assessment of changes in the QIP review process through 30- to 45-minute online interviews with service providers post-intervention, focusing on how the Toolkit assisted with the review process. Interviews were, therefore, only conducted with service providers in the intervention group. Additional outcomes were collected via online surveys at baseline and post-intervention on self-ratings of awareness and knowledge of *Munch & Move* practices; NQS; prioritizing nutrition and physical activity practices; and identifying priority areas.

### Procedures

After consultation with New South Wales Family Day Care Association stakeholders, a target sample of 10 to 12 service providers (and their educators) was established. This was considered feasible within the constraints of engaging the family day care sector. Previous research conducted by our institution also identified that this was a difficult sector to recruit due to their working schedules.

Participants were randomized 1:1 using a permuted block design with a computerized random number generator. A data manager with no involvement in the study conducted the randomization. The participants and researcher implementing the intervention (G.T.) were not blinded to the allocation due to the nature of the intervention.

### Data Analysis

Analyses followed the intention-to-treat approach and were performed in jamovi version 2.4.12 (the jamovi project, 2024). Null hypotheses were tested at the .05 significance level (two-sided). Missing data were addressed using complete case analysis, suitable when missing data are below 5% (Jakobsen et al., 2017).

To examine the difference in primary outcome between the two groups (intervention vs. control), a repeated measures analysis of variance (ANOVA) was conducted. As the primary outcome focused on the QIP, only service providers data were used in the analysis. No adjustments were made for variables due to the small sample size and no baseline differences in characteristics. Analyses were repeated for each checklist section.

Secondary outcomes (self-ratings of awareness and knowledge of *Munch & Move* practices; NQS; prioritizing nutrition and physical activity practices; identifying priority areas) were analyzed using all participant data. Linear mixed models accounted for the random effect of groups (educators grouped under service providers). Adjustments for educational attainment (certificate III and diploma vs. undergraduate degree), nutrition and physical activity training (last completed within the last 12 months vs. more than 12 months ago), and main home language (English vs. other) were included in the models.

Qualitative data for the outcome of change in the QIP review process used service provider data from the intervention group only. Data obtained were grouped into themes and analyzed using NVivo version 12.7.

## Results

**Figure 1:**
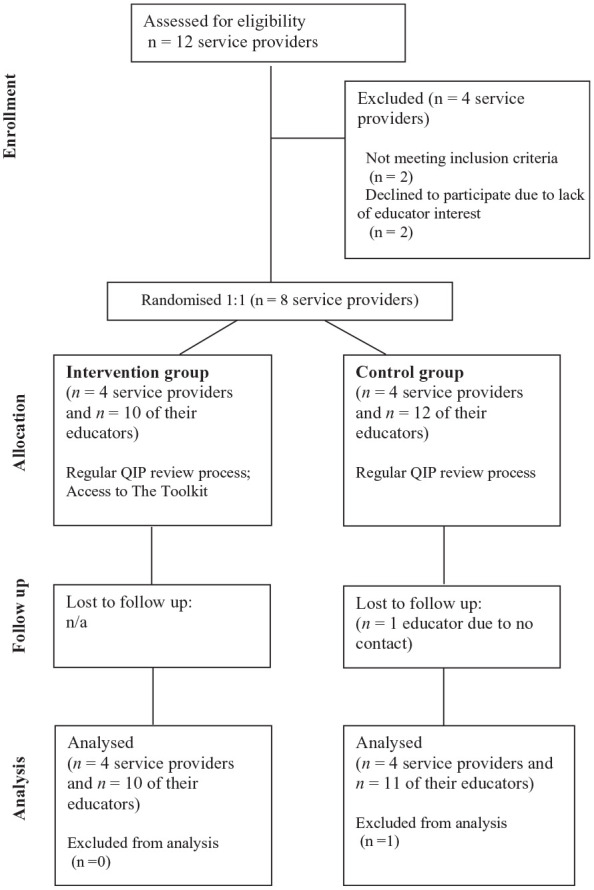
Flow of Participants Through the Study. One Educator Lost to Follow Up in the Control Group After Study Completion. All Other Participants Included in Final Analysis (*n* = 30)

Of the 118 New South Wales service providers identified (Australian Children’s Education & Care Quality Authority, n.d.), 12 expressed interest. Four were excluded: two were not undergoing QIP reviews and two with insufficient educator interest. Eight service providers were enrolled and randomized. The enrolled service providers recruited 22 educators, resulting in 30 participants. Only one educator was lost to follow-up. The participant flowchart is depicted in Figure 1.

Almost all participants (96.7%) were female. The mean age was 48 years (*SD* = 10). The most common home language was English (60%). Most participants (63%) held a Diploma in Early Childhood Education and Care as their highest qualification. One third (33%) had last completed training in healthy eating or physical activity more than 2 years ago.

Fourteen participants (four service providers and 10 of their educators) were allocated to the intervention group. All engaged with at least one Toolkit feature. Process outcomes (e.g., metrics of use) were not defined due to the variability in QIP review processes. Data from the website back-end showed that there were 116 questionnaires submitted by educators (average of 11 questionnaires per educator), 66 self-assessment reflections submitted by educators (average of six reflections per educator), and 21 action plans submitted by service providers (average of five action plans per service provider). There were no reported privacy breaches.

All participants rated the Toolkit as “good” or “excellent” for accessibility, usefulness, and interest in future use. One participant rated their understanding of the information as “poor,” potentially due to limited computer literacy, as indicated by the open-text feedback. Six participants provided the optional open-text feedback. The feedback related to the benefits of the tool and offering minor technical suggestions.

Table 1 presents the intervention effects for the change in the quality of the QIP in relation to nutrition and physical activity practices. Assumption testing showed normal data distribution. Post hoc Tukey test showed a statistically significant increase in QIP score within the intervention group between baseline and follow-up, with a mean score difference of –8.500 (out of 17) (95% confidence interval [CI] = [–11.49, –5.51]; *p* = .002). There was a statistically significant difference at follow-up between the intervention and the control group with a mean score difference of 7.75 (95% CI = [4.54, 10.96]; *p* = .004).

**Table 1:** Intervention Effect on Change in Quality of Quality Improvement Plan (Primary Outcome) Assessed Using Post Hoc Comparisons Following Repeated Measures ANOVA (*n* = 4 in Each Treatment Arm)

Post hoc comparisons—Time × Treatment									
Comparison									
Time	Treatment		Time	Treatment	Mean difference	SE	df	t	p_tukey_
Baseline	Intervention	-	Baseline	Control	0.75	2.16	6.00	0.35	.98
		-	Follow-Up	Intervention	–8.50	1.22	6.00	–6.94	.002
		-	Follow-Up	Control	–0.75	1.79	6.00	–0.42	.97
	Control	-	Follow-Up	Intervention	–9.25	1.79	6.00	–5.18	.008
		-	Follow-Up	Control	–1.50	1.22	6.00	–1.23	.64
Follow-Up	Intervention	-	Follow-Up	Control	7.75	1.31	6.00	5.89	.004

The same analyses were repeated for each checklist section. For the first section examining QIP components, the post hoc Tukey test showed a statistically significant difference at follow-up between the intervention and the control group with a mean score difference of 3.25 (out of 9) (95% CI = [1.41, 5.09]; *p* = .02). Similarly, the post hoc test for the second section examining alignment with *Munch & Move* practices showed a statistically significant difference at follow-up between the intervention and the control group with a mean score difference of 4.50 (out of 8) (95% CI = [2.56, 6.44]; *p* = .005).

For secondary outcomes, factors accounted for in the linear mixed models were educational attainment, nutrition and physical activity training and main home language. Random components analyses focused on effects of educators grouped under service providers.

There was a statistically significant group-by-time interaction for awareness and knowledge of *Munch & Move* practices (–1.08; 95% CI = [–1.42, –0.73]; *p* < .001) and NQS (–0.51; 95% CI = [–0.82, -0.20]; *p* = .003), and confidence in identifying priority areas (–0.68; 95% CI = [–0.95, –0.41]; *p* < .001), whereby the intervention group improved their awareness and knowledge in all areas at follow-up compared with baseline and the control group. No statistically significant group-by-time interaction was found for the importance of promoting good nutrition and physical activity behaviors (*p* = .89). Random components analyses indicated high correlation between educators within the same service provider for self-ratings of awareness and knowledge of *Munch & Move* practices and confidence in identifying priority areas (intraclass correlation coefficient [ICC]= .38 and .26, respectively).

Three main themes were identified from the qualitative data obtained from service providers in the intervention group (*n* = 4) in relation to how the Toolkit changed their QIP review process.

The theme of “supporting educators” highlighted how the Toolkit allowed educator data to be included in the QIP where previously this had not been the case, as well as providing useful resources for educator professional development. For example, it was highlighted that the tool allowed for direct input from educators and this was helpful as educators were not often involved in the QIP review process.

The theme of “promoting nutrition and physical activity practices” referred to the Toolkit highlighting *Munch & Move* priority areas and identifying gaps in educator knowledge. For example, data from the educator questionnaire highlighted to a participating service provider that there were particular *Munch & Move* areas they previously thought were being met by their educators; however, the results suggested there may be a lack of knowledge among educators.

The theme of “ease of use and convenience” referred to benefits of web-based tool usage such as easy access to ongoing data input from educators. For example, a participating service provider commented that their current process of accessing educator critical reflections was through emails and it was much easier to have all educator performance data on the one platform (the Toolkit).

Three service providers suggested refining the tool to cover all seven NQS quality areas alongside nutrition and physical activity. Technical suggestions included incorporating data filters and viewing all action plan submissions on one page. Technical issues during the study were minor and none affected participants from using the tool appropriately. One participant highlighted a new QIP process introduced by the New South Wales Department of Education after the study period and recommended considering this in tool refinement.

## Discussion

This study evaluated the acceptability of a web-based tool designed specifically for family day care and its effectiveness in incorporating nutrition and physical activity practices into quality improvement planning. We found a significant difference in QIP quality (in relation to the incorporation of nutrition and physical activity practices) between the intervention and control groups at follow-up. The intervention demonstrated positive group-by-time outcomes for self-rated awareness and knowledge of *Munch & Move* and NQS, and confidence in identifying priority areas. There was no significant group-by-time interaction for self-rating of the importance of promoting nutrition and physical activity behaviors. We found a high correlation among educators with the same service provider for self-ratings of awareness and knowledge on *Munch & Move* practices and confidence in identifying nutrition and physical activity priority areas.

Service providers using the Toolkit alongside their regular QIP review process achieved higher quality QIPs in terms of incorporation of nutrition and physical activity practices. Many quality improvement initiatives that focus on guiding specific components of a good quality action plan have found similar positive outcomes. For example, a Colombian hospital found a significant increase in hand hygiene adherence through a multimodal action plan addressing staff education, accountability, product selection and accessibility, and organizational culture (Johnson et al., 2014). Another project implementing a standardized seizure action plan based on plan-do-study-act cycles led to an improvement in key elements of caregiver education (Neville et al., 2020). The “action effect” method is a strong framework to guide the implementation of quality improvement initiatives by aligning planned interventions with an overall improvement aim and identifying appropriate outcome measures (Reed et al., 2014). In the present study, the Toolkit provided a structured approach for service providers to identify gaps in their educator practices and develop an action plan that also addressed relevant *Munch & Move* practices and NQS.

Positive group-by-time outcomes were found regarding self-rated awareness and knowledge of *Munch & Move* practices and NQS, and confidence in identifying nutrition and physical activity priority areas. This is valuable given previous inconclusive results of family day care health interventions on children’s physical activity and weight, and it is suggested that future interventions involve stakeholder input and enhance service providers’ positions as role models (Vorage et al., 2024). A study in Australian family day care found that few schemes had comprehensive breastfeeding and screen time policies, and educators only implemented an average of 64% of recommended healthy practices (Lum et al., 2021). The Toolkit in the present study, co-developed with key stakeholders, focused on change at a provider level to better support children’s healthy behaviors. This is important not only because of the co-design process, but it has been shown that professional development is more effective when family day care professionals feel empowered and can engage in continuous quality improvement rather than completing training simply to meet licensing requirements (Lanigan, 2011).

No significant group-by-time interaction was found for self-rating of the importance of promoting good nutrition & physical activity behaviors. This was expected as the small sample size likely represented those who were most engaged in the research topic. Child care providers generally perceive that they play an important role in childhood obesity prevention (Rosenthal et al., 2013). However, this is not necessarily reflected in practice. For example, a study investigating food offered to children below 5 years of age in U.K. day care found that although the majority of providers felt a responsibility for promoting the health of children, only one third of day care centers provided a fruit or vegetable for lunch (Moore et al., 2005). Therefore, it is important when developing interventions to consider not only the professional’s beliefs in the importance of nutrition and physical activity but to also ensure that professionals are equipped to translate this into practice.

There was a high correlation between educators within the same service provider for self-ratings of awareness and knowledge of *Munch & Move* practices and confidence in identifying nutrition and physical activity priority areas. This is not surprising as family day care educators are resourced, supported, and monitored by the service provider coordination unit (Ishimine & Tayler, 2012). It has been shown that service providers trained in the *Munch & Move* program were more likely to offer healthy eating and physical activity professional development to their educators (Kerr et al., 2022). This highlights the importance of interventions targeting service providers as they can support their educators in appropriate training to improve educator practices. It has been demonstrated that children in family child care homes in which more of best practices were implemented had a higher diet quality (Jiang et al., 2023). This suggests that it is worthwhile to improve the implementation and adoption of the *Munch & Move* program across family day care and highlights the importance of engaging with not only educators but also with service providers and acknowledging their role in providing support to their educators.

Recruitment was challenging despite state-wide efforts and collaboration with Local Health Districts. The unique family day care setting, with educators working from their home and beyond usual business hours, may preclude research engagement. Further work is required to improve engagement with this sector. The Toolkit was described as a tool to promote healthier nutrition and physical activity practices that appeared to have attracted participants who valued this. Further work should explore adaptations of the tool for hard-to-reach populations.

This study has several limitations. A longer recruitment or follow-up period may have increased participant numbers; however, this was not feasible for this project. Self-rated measures may have involved cognitive bias. The small and state-wide sample limits the generalizability of results. No participants resided in low socioeconomic areas and the tool may not have been as effective for hard-to-reach populations. A cost-effectiveness analysis was not within the study’s scope.

## Conclusion

The Toolkit significantly improved QIP quality in relation to the incorporation of nutrition and physical activity practices, awareness and knowledge of *Munch & Move* practices, awareness and knowledge of NQS, and confidence in identifying priority areas. This is the first-known tool designed for family day care to promote healthier practices through an existing quality improvement process. The Toolkit could be scaled to cover all seven NQS areas or adapted for hard-to-reach populations. There are several opportunities for integration into practice, including delivery of the tool as part of support programs or training.

## Implications for Practice

Health promotion practitioners should consider embedding the web-based tool into professional development frameworks and integrating it into ongoing training programs for educators. This approach would enhance educators’ capacity to incorporate nutrition and physical activity practices into quality improvement planning. Refinements to the tool could include features to foster social support among educators, such as online discussion forums or peer networking opportunities, which may further enhance its impact. Offering multilingual options would improve accessibility, ensuring broader reach among diverse populations. In addition, aligning the tool’s structure with established frameworks and best practices in early childhood nutrition and physical activity would support its adoption and consistent implementation within the sector.

## Supplemental Material

sj-docx-1-hpp-10.1177_15248399251328360 – Supplemental material for Web-Based Tool for Australian Family Day Care to Promote Healthy Lifestyles: Randomized Controlled Trial Supplemental material, sj-docx-1-hpp-10.1177_15248399251328360 for Web-Based Tool for Australian Family Day Care to Promote Healthy Lifestyles: Randomized Controlled Trial by Georgie Tran, Bridget Kelly, Sarah T. Ryan, Megan Hammersley, Erin Kerr, Jennifer Norman, Mel Leedham, Cecilia Vuong, Karen Wardle and Anthony Okely in Health Promotion Practice

sj-docx-2-hpp-10.1177_15248399251328360 – Supplemental material for Web-Based Tool for Australian Family Day Care to Promote Healthy Lifestyles: Randomized Controlled Trial Supplemental material, sj-docx-2-hpp-10.1177_15248399251328360 for Web-Based Tool for Australian Family Day Care to Promote Healthy Lifestyles: Randomized Controlled Trial by Georgie Tran, Bridget Kelly, Sarah T. Ryan, Megan Hammersley, Erin Kerr, Jennifer Norman, Mel Leedham, Cecilia Vuong, Karen Wardle and Anthony Okely in Health Promotion Practice
