# Supplementary material for: Web-Based Tool for Australian Family Day Care to Promote Healthy Lifestyles: Randomized Controlled Trial
Source: Health Promot Pract. 2025 Mar 30;27(2):269–76. doi: 10.1177/15248399251328360 (PMC12913684; doi:10.1177/15248399251328360)
Supplement: sj-docx-1-hpp-10.1177_15248399251328360 – Supplemental material for Web-Based Tool for Australian Family Day Care to Promote Healthy Lifestyles: Randomized Controlled Trial [file sj-docx-1-hpp-10.1177_15248399251328360.docx]

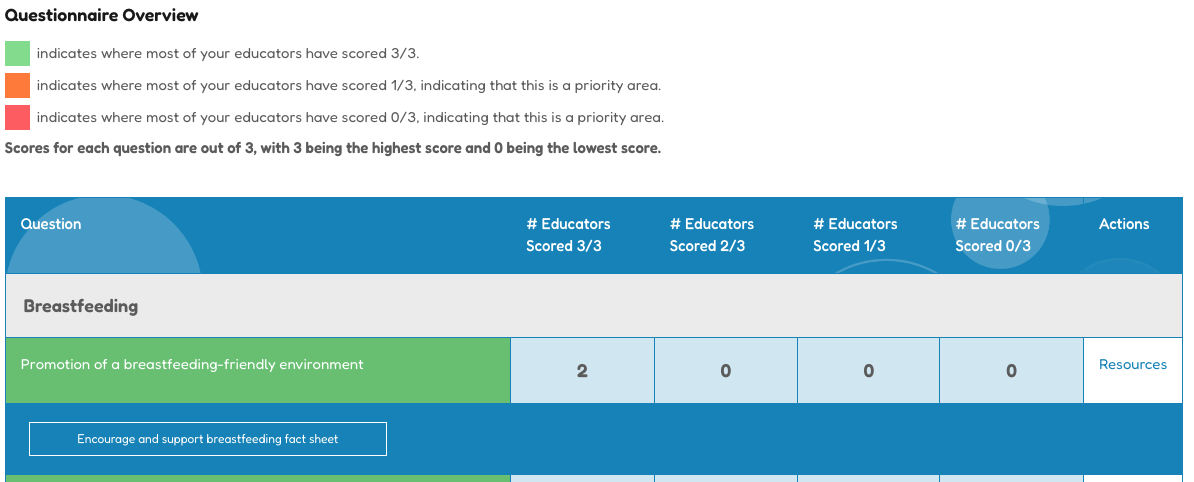

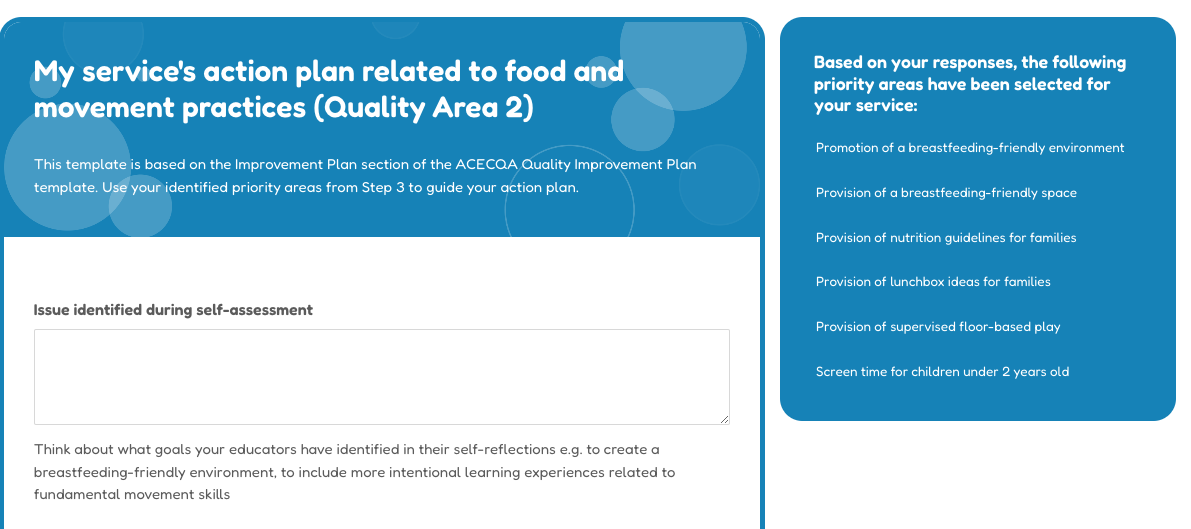

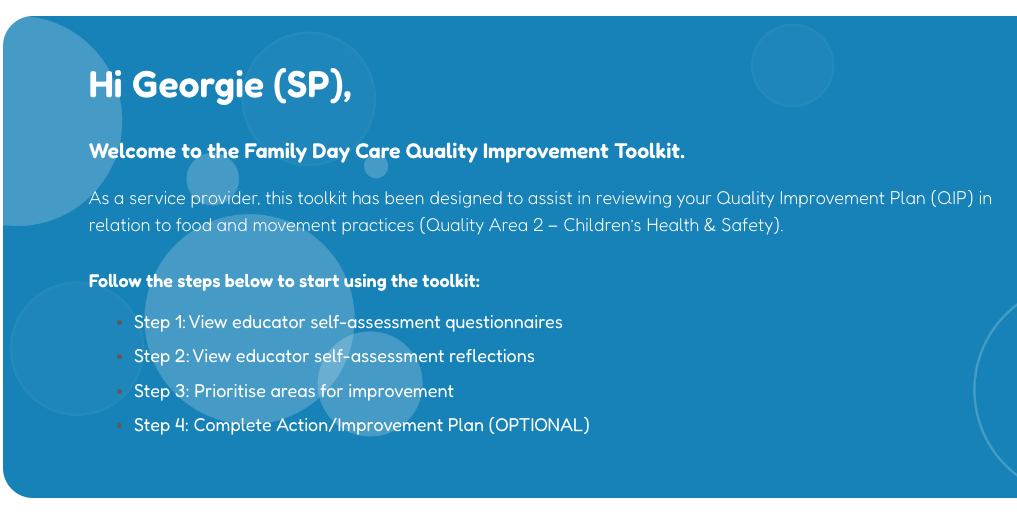
**Supplementary File 1.** Screenshots of the login pages of the Toolkit.

Screenshots from the *Family Day Care Quality Improvement Toolkit* showing examples from the website using a service provider login (i.e. home page after login, tailored Action Plan template, and overview of educator questionnaire performance).


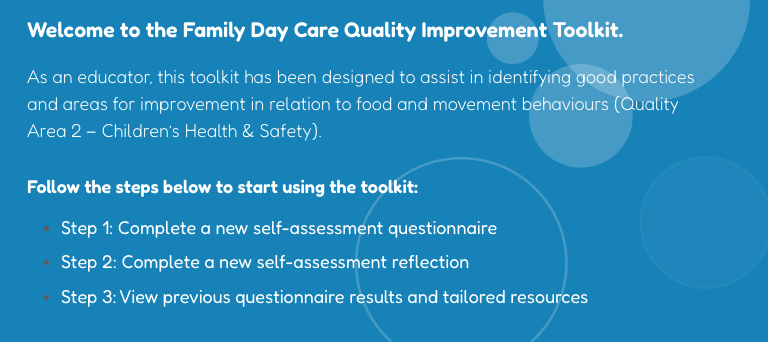


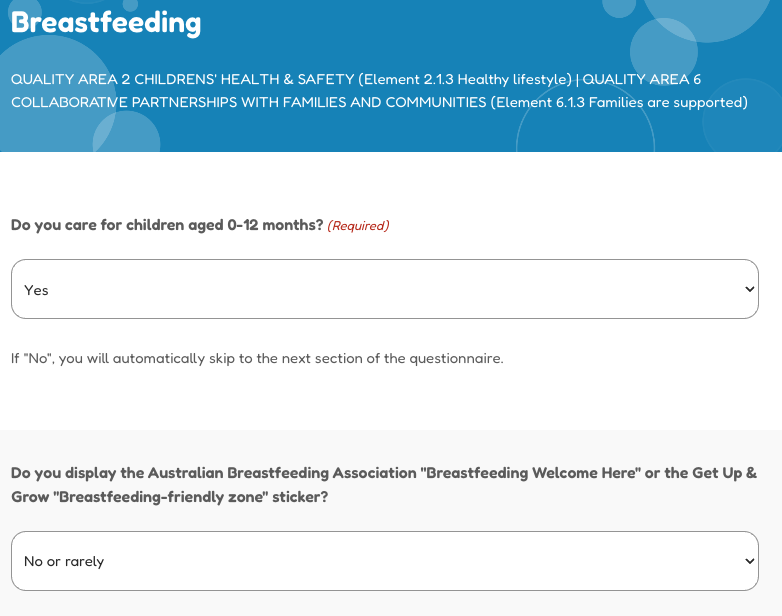


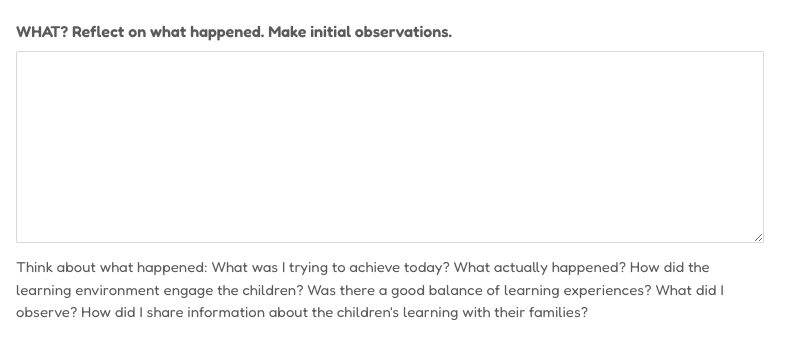


Screenshots from the *Family Day Care Quality Improvement Toolkit* showing examples from the website using an educator login (i.e. home page after login, questionnaire to identify priority areas, and part of the guided self-reflection).
