# Supplementary material for: Web-Based Tool for Australian Family Day Care to Promote Healthy Lifestyles: Randomized Controlled Trial
Source: Health Promot Pract. 2025 Mar 30;27(2):269–76. doi: 10.1177/15248399251328360 (PMC12913684; doi:10.1177/15248399251328360)
Supplement: sj-docx-2-hpp-10.1177_15248399251328360 – Supplemental material for Web-Based Tool for Australian Family Day Care to Promote Healthy Lifestyles: Randomized Controlled Trial [file sj-docx-2-hpp-10.1177_15248399251328360.docx]

**Supplementary File 2.** Quality Improvement Plan Checklist

|  | QIP Component Checklist | Yes  (score = 1) | No  (score = 0) |
| --- | --- | --- | --- |
| 1 | **Addressing National Quality Standard** |  |  |
|  | Are practices related to nutrition mentioned under Quality Area 2 of the QIP? |  |  |
|  | Are practices related to physical activity and sedentary behaviour mentioned under Quality Area 2 of the QIP? |  |  |
| 2 | **Identifying priority areas** |  |  |
|  | Are any practices related to nutrition identified as an area for improvement? |  |  |
|  | Are any practices related to physical activity and sedentary behaviour identified as an area for improvement? |  |  |
|  | Have any *Munch & Move* practices/key areas been identified as an area for improvement? |  |  |
| 3 | **Creating reasonable action/improvement plans** |  |  |
|  | Are there plans in place to address areas of improvement related to nutrition? |  |  |
|  | Are there plans in place to address areas of improvement related to physical activity and sedentary behaviour? |  |  |
|  | Are action/improvement plans clearly linked to specific Quality Areas of the National Quality Standard? |  |  |
| 4 | **Educator involvement** |  |  |
|  | Are staff (educators) self-assessments of their own practices included in the service’s QIP? |  |  |

| QIP *Munch & Move* Practices Checklist | Yes, this practice was both mentioned in the QIP and aligned with good practice* (score = 2) | Yes, this practice was mentioned only (score = 1) | No, this practice was not mentioned nor aligned with good practice* (score = 0) |
| --- | --- | --- | --- |
| Does the QIP mention and/or align with good practices* related to the *Munch & Move* practice “Encouraging Healthy Eating”? |  |  |  |
| Does the QIP mention and/or align with good practices* related to the *Munch & Move* practice “Including Daily Physical Activity”? |  |  |  |
| Does the QIP mention and/or align with good practices* related to the *Munch & Move* practice “Putting In Place Policies”? |  |  |  |
| Does the QIP mention and/or align with good practices* related to the *Munch & Move* practice “Educating and Monitoring”? |  |  |  |
| TOTAL SCORE |  | | |

** Good practice will be defined by the “Munch & Move Program Adoption Indicator (Practice)” internal document for health promotion officers. This document was used in the development of the Questionnaire and related educational resources in the tool, therefore utilising this document to define good practice will also ensure alignment with the resources provided in the tool.*
